# Beyond Bone Density Alone: Opportunistic Identification of Vertebral Compression Fractures in Breast Cancer Survivors Using Artificial Intelligence-Derived Vertebral Bone Density and Paraspinal Muscle–Fat Metrics

**DOI:** 10.3390/jcm15166283

**Published:** 2026-08-13

**Authors:** Chengxin Wan, Lingquan Kong, Jie Hao, Bin Lu, Chao Wu, Miao Wei, Zhiwei Zhang, Beibei Gong, Fajin Lv

**Affiliations:** 1 Department of Radiology, The First Affiliated Hospital of Chongqing Medical University, Chongqing 400016, China; chengxinwan@hospital.cqmu.edu.cn (C.W.);; 2 Department of Breast and Thyroid Surgery, The First Affiliated Hospital of Chongqing Medical University, Chongqing 400016, China; 3 Department of Orthopedics, The First Affiliated Hospital of Chongqing Medical University, Chongqing 400016, China

**Keywords:** artificial intelligence, breast cancer, computed tomography, vertebral compression fracture, volumetric bone mineral density, paraspinal intermuscular adipose tissue

## Abstract

**Background/Objectives**: This study aimed to evaluate whether artificial intelligence-derived vertebral volumetric bone mineral density (AI-vBMD) and paraspinal intermuscular adipose tissue (IMAT) ratio from routine computed tomography (CT) could identify moderate-to-severe vertebral compression fractures (VCFs) in breast cancer survivors, and whether paraspinal IMAT ratio and routinely available clinical variables improved diagnostic performance. **Methods**: This retrospective study included 275 women with breast cancer who underwent routine non-contrast CT and lumbar quantitative computed tomography (QCT). Hounsfield unit-derived volumetric bone mineral density (HU-vBMD) was derived using a QCT-referenced HU-to-vBMD conversion equation, whereas AI-vBMD and paraspinal IMAT ratio were extracted using automated software. Moderate-to-severe VCF was defined as Genant grade ≥ 2. Agreement with QCT-vBMD was assessed using correlation, intraclass correlation coefficient (ICC), and Bland–Altman analysis. Model discrimination was evaluated using receiver operating characteristic analysis and DeLong tests. **Results**: Moderate-to-severe VCF was present in 75 patients (27.3%). HU-vBMD and AI-vBMD showed excellent agreement with QCT-vBMD (ICC, 0.978 and 0.987, respectively). AI-vBMD outperformed HU-vBMD for identifying VCFs (AUC, 0.738 vs. 0.714; *p* < 0.001). IMAT ratio showed comparable standalone discrimination to AI-vBMD (AUC, 0.760 vs. 0.738; *p* = 0.604). Adding IMAT ratio to AI-vBMD improved discrimination (AUC, 0.786 vs. 0.738; *p* = 0.038). The full model incorporating clinical covariates achieved the highest AUC (0.828; 95% CI, 0.778–0.878). **Conclusions**: AI-vBMD and paraspinal IMAT ratio automatically extracted from routine CT improved the diagnostic assessment of prevalent moderate-to-severe VCFs in breast cancer survivors. This study supports an automated CT-based approach that integrates vertebral bone density and paraspinal muscle–fat information for opportunistic identification of clinically relevant VCFs.

## 1. Introduction

With improved breast cancer survival, skeletal fragility has become an increasingly important component of survivorship care [1,2]. Women with breast cancer are particularly vulnerable to bone loss owing to aging, menopause, treatment-related endocrine changes, and adjuvant therapies that may accelerate skeletal deterioration [3,4]. Vertebral compression fractures (VCFs) are particularly relevant in this setting because they are frequently underrecognized on routine imaging, may occur with minimal symptoms, and can indicate clinically meaningful skeletal vulnerability [5,6]. Among these, moderate-to-severe VCFs, commonly defined as Genant grade ≥ 2, are more likely to reflect substantial vertebral structural compromise and therefore represent an important imaging endpoint for clinical assessment of prevalent vertebral fragility [7].

Dedicated bone assessment remains essential for evaluating skeletal health. Dual-energy X-ray absorptiometry is widely used in clinical practice, whereas quantitative computed tomography (QCT) provides volumetric bone mineral density (vBMD) and enables trabecular bone assessment [8]. However, dedicated bone examinations are not always performed in parallel with oncologic surveillance. In contrast, many patients with breast cancer routinely undergo CT examinations as part of clinical care. These images contain quantitative information about vertebral bone and paraspinal soft tissues that is often not fully used. The practical challenge is therefore not merely to obtain additional bone tests, but to extract actionable skeletal information from examinations that patients already undergo [9,10].

Opportunistic CT-based bone assessment has emerged as a pragmatic strategy to bridge this gap. Vertebral attenuation measured in Hounsfield units (HU) can be converted to a QCT-referenced vBMD scale, improving interpretability beyond raw attenuation values [11,12]. Automated artificial intelligence (AI)-based tools may further standardize vertebral recognition, segmentation, vBMD estimation, and extraction of paraspinal muscle–fat features from routine CT [13,14]. Nevertheless, vertebral fragility in breast cancer survivors is unlikely to be explained by bone density alone. Treatment-related endocrine changes, adiposity, and altered body composition may affect the paraspinal muscle–fat compartment, which is biomechanically coupled to spinal stability [15,16]. Although prior studies have linked paraspinal muscle degeneration and combined bone-muscle imaging features to vertebral fracture status or fracture-related vulnerability [15,17], whether this bone-muscle framework translates to breast cancer survivors undergoing routine CT remains insufficiently defined.

Evidence remains limited on whether CT-based bone density and AI-derived paraspinal muscle–fat metrics provide complementary value for identifying clinically relevant VCFs in women with breast cancer. In particular, the relationship among QCT-referenced HU-vBMD, AI-derived vBMD, AI-derived paraspinal intermuscular fat ratio, and Genant-defined moderate-to-severe VCF has not been fully clarified within a unified framework. Therefore, this study aimed to evaluate the agreement of HU-vBMD and AI-vBMD with QCT-vBMD and to determine the diagnostic performance and incremental value of CT-derived bone density and AI-derived paraspinal IMAT ratio for identifying Genant grade ≥ 2 VCFs in women with breast cancer.

## 2. Materials and Methods

This retrospective, non-interventional study used de-identified data and complied with the Declaration of Helsinki. The Ethics Review Committee of the First Affiliated Hospital of Chongqing Medical University approved the protocol and waived the requirement for written informed consent (approval No. CYFYY276-02). Reporting followed the STROBE recommendations and the STARD checklist where applicable. STROBE was applied to the observational cross-sectional components of the study, whereas STARD was applied to the diagnostic accuracy analyses where applicable. The STROBE and STARD checklists are provided as Supplementary Material.

### 2.1. Participants

**Figure 1:**
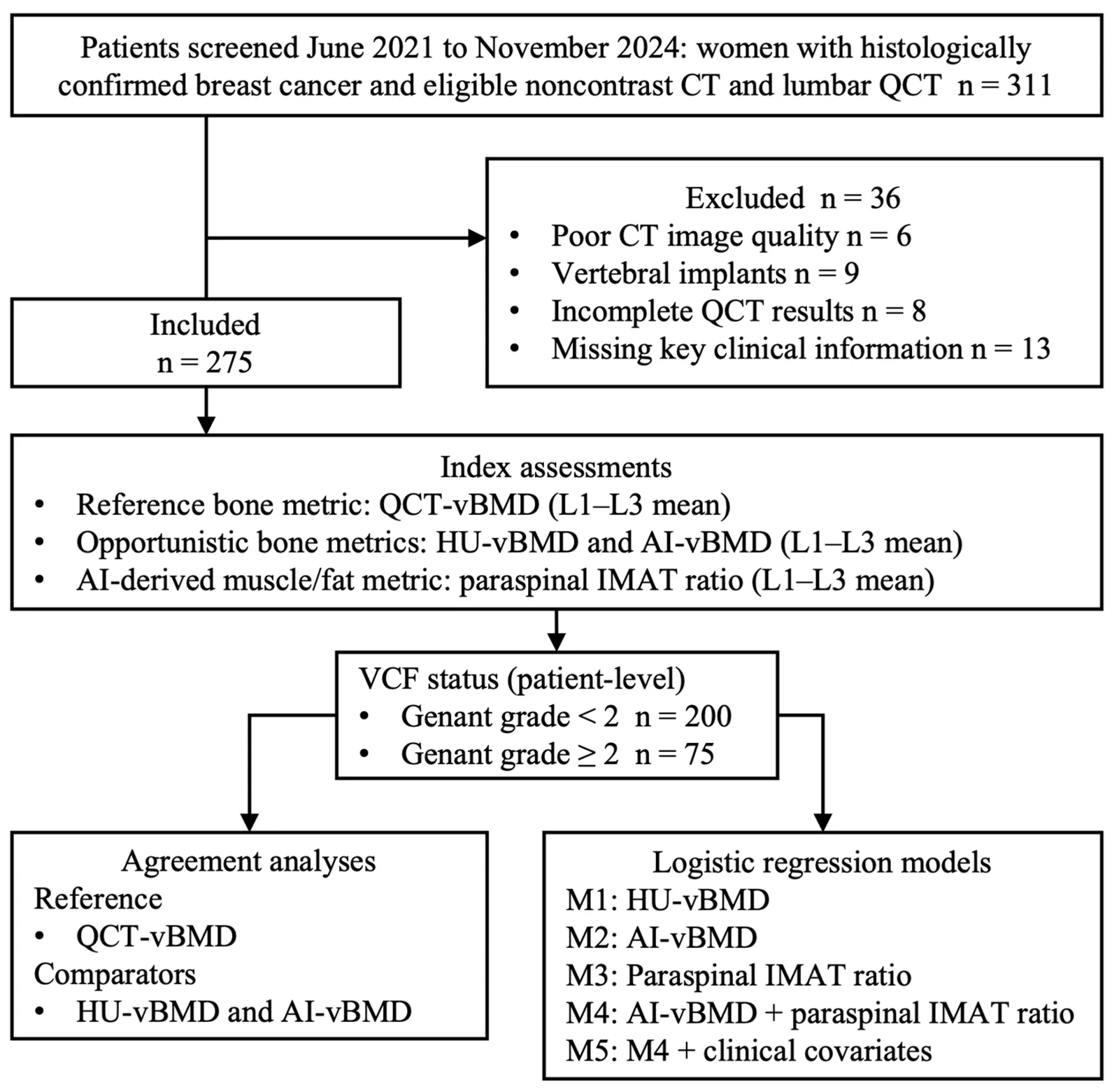
Patient selection and analytic cohort. Consecutive women with histologically confirmed breast cancer who underwent routine non-contrast CT with evaluable L1–L3 coverage and lumbar QCT within 7 days were screened. Patients were excluded according to predefined eligibility criteria. The final analytic cohort included 275 women, of whom 75 had moderate-to-severe VCFs, defined as Genant grade ≥ 2. Abbreviations: CT, computed tomography; QCT, quantitative computed tomography; VCF, vertebral compression fracture.

This retrospective study included consecutive adult women with histologically confirmed breast cancer who underwent routine non-contrast CT with evaluable L1–L3 coverage and lumbar QCT within 7 days at a single tertiary referral center between June 2021 and November 2024. Patients were excluded if the index vertebrae were affected by metastasis or other malignant infiltration, if prior vertebral instrumentation or augmentation was present, if CT image quality was non-diagnostic, or if QCT or key clinical data were incomplete. Because iodinated contrast can increase vertebral attenuation and bias CT-based bone measurements, only true unenhanced CT series were included [18,19]. The study flow and final analytic cohort are shown in Figure 1. A total of 311 women with histologically confirmed breast cancer undergoing routine follow-up or clinical CT evaluation were screened. Neither current treatment status nor metastatic disease outside the spine was an exclusion criterion. Of these, 36 were excluded because of poor CT image quality, vertebral implants, incomplete QCT results, or missing key clinical information, leaving 275 patients in the final analytic cohort. Adjuvant endocrine therapy was recorded as a binary variable according to documented treatment exposure before or at the time of CT examination. Because detailed information on endocrine regimen, treatment duration, adherence, and cumulative exposure was not consistently available, endocrine therapy was modeled as a yes/no covariate rather than by specific drug class. No a priori sample size calculation was performed because this was a retrospective study based on all consecutive eligible patients during the study period.

### 2.2. CT Acquisition

All CT examinations were performed on a dual-source CT system (SOMATOM Force, Siemens Healthineers, Erlangen, Germany) using a standard non-contrast thoracoabdominal protocol routinely applied in oncologic imaging practice. Scan parameters were as follows: detector collimation, 192 × 0.6 mm; tube voltage, 120 kVp; automatic tube current modulation; and gantry rotation time, 0.25 s. Images were reconstructed with a medium soft-tissue kernel (Br40) at a slice thickness of 1.0 mm and an interval of 1.0 mm, using a 512 × 512 matrix and a 32 cm field of view. All analyzed series were non-contrast acquisitions. Images were reviewed on the institutional picture archiving and communication system.

### 2.3. QCT Reference Standard

QCT-derived volumetric bone mineral density (QCT-vBMD, mg/cm^3^) served as the reference standard for agreement analyses. Lumbar trabecular vBMD was obtained at L1 to L3 using asynchronous calibration and QCT Pro analysis software (Model 4, Version 6.1; Mindways Software, Inc., Austin, TX, USA), under routine quality assurance procedures including daily phantom-based quality checks and periodic scanner calibration, following institutional practice. The primary QCT metric was the mean QCT-vBMD across L1 to L3. QCT-vBMD was obtained using QCT Pro with asynchronous calibration.

### 2.4. HU Measurement and HU-Derived vBMD

Trabecular attenuation was measured at L1, L2, and L3 on non-contrast CT images using a 1 mm bone window. For each vertebra, an oval region of interest was placed along the inner margin of the cortical bone on the upper, mid, and lower portions of the vertebral body, while avoiding the basivertebral venous plexus, focal lesions, and obvious fracture lines. The mean HU for each vertebra was defined as the average of the three section measurements, and the L1 to L3 mean HU was calculated as the patient-level HU metric.

HU vBMD (HU-vBMD, mg/cm^3^) was computed by fitting a linear regression model between the L1 to L3 mean HU and the corresponding QCT-vBMD. The resulting equation was then applied to the HU values to generate HU-vBMD for each participant.

### 2.5. AI-Derived vBMD and Paraspinal Muscle–Fat Metrics from Routine CT

AI-derived vertebral volumetric bone mineral density (AI-vBMD) was obtained using commercially available, fully automated CT analysis software developed by Huiying Medical Technology (Beijing, China; version 2.11.0), which does not require a dedicated external calibration phantom during routine CT acquisition. The software automatically performs vertebral recognition, segmentation, and quantification of trabecular attenuation, and outputs vertebral vBMD in physical units (mg/cm^3^) through an internal calibration algorithm. Detailed information on the calibration loop, including whether internal reference tissues such as blood pool, fat, or other in-field tissues are sampled to normalize scanner- or protocol-related HU variability, was not disclosed by the vendor and was not independently accessible to the investigators. Similar automated CT-based approaches that do not require concurrent patient–phantom scanning have been validated for opportunistic vertebral BMD assessment in prior studies [12,13,20]. In addition, this commercial software platform has recently been used in an independent clinical study of opportunistic osteoporosis assessment on routine CT [21].

The same software also automatically outputs paraspinal muscle area (mm^2^) and paraspinal intermuscular adipose tissue (IMAT) area (mm^2^) at the axial mid-vertebral levels of L1, L2, and L3. The paraspinal muscle compartment included the bilateral erector spinae and multifidus muscles and the corresponding intermuscular fat spaces. The primary AI-derived bone metric was the mean AI-vBMD across L1–L3. For muscle–fat analysis, the vertebral-level paraspinal IMAT ratio was calculated as paraspinal IMAT area divided by paraspinal muscle area at each level, and the primary muscle–fat metric was the mean paraspinal IMAT ratio across L1–L3. AI-derived measurements used in this study were obtained directly from the automated software output without manual ROI delineation or manual quantitative adjustment.

### 2.6. Vertebral Compression Fracture Assessment

Vertebral compression fractures were assessed on sagittal multiplanar reformatted CT images covering T4–L5. Two musculoskeletal radiologists (Z.W.Z. and B.B.G., with 4 and 9 years of experience, respectively) independently graded each vertebra using the Genant visual semiquantitative method (grades 0–3) [7]. The readers were blinded to HU-vBMD, QCT-vBMD, AI-derived measurements, model outputs, and clinical information. Cases were reviewed in a computer-generated random order, and window level and width could be adjusted as needed.

For patient-level analysis, the highest vertebral grade was recorded. Genant grades 2–3 were defined as moderate-to-severe vertebral compression fractures and served as the primary imaging endpoint. Fractures suspicious for metastatic involvement, including cortical destruction, pedicle involvement, paravertebral soft-tissue mass, or known metastatic involvement at the same vertebral level, were excluded according to predefined criteria. Discrepant cases were re-reviewed after a washout period of at least 4 weeks, and the consensus grade was used as the final reference standard.

### 2.7. Statistical Analysis

All statistical analyses were performed using R software (version 4.5.2; R Foundation for Statistical Computing, Vienna, Austria). Continuous variables were summarized as mean ± standard deviation or median with interquartile range, and categorical variables as counts and percentages. Group comparisons between patients with Genant grade < 2 and those with Genant grade ≥ 2 were performed using the Student *t* test or Wilcoxon rank-sum test for continuous variables and the chi-square test or Fisher exact test for categorical variables, as appropriate. Analyses were performed using complete-case data. All tests were two-sided, and *p* values < 0.05 were considered statistically significant.

Agreement between CT-derived BMD metrics and QCT-vBMD was assessed using Pearson correlation coefficients, intraclass correlation coefficients (two-way random-effects model, absolute agreement, single measurement), and Bland–Altman analyses. HU-vBMD was generated from a cohort-specific QCT-referenced HU-to-vBMD linear regression equation; therefore, agreement involving HU-vBMD was interpreted as within-cohort calibration performance. To identify prevalent moderate-to-severe VCFs, defined as Genant grade ≥ 2, prespecified logistic regression models were constructed: M1, HU-vBMD alone; M2, AI-vBMD alone; M3, paraspinal IMAT ratio alone; M4, AI-vBMD plus IMAT ratio; and M5, M4 plus age, body mass index, adjuvant endocrine therapy, and radiotherapy. Adjuvant endocrine therapy and radiotherapy were modeled as binary covariates based on medical record documentation. Continuous predictors were standardized as z scores before modeling, and odds ratios with 95% confidence intervals were reported. Collinearity diagnostics for M5 are provided in Supplementary Table S4.

Model discrimination was evaluated using receiver operating characteristic curves and AUCs with 95% confidence intervals. Prespecified paired DeLong comparisons included M1 versus M2, M1 versus M3, M2 versus M3, M2 versus M4, and M4 versus M5. Sensitivity, specificity, positive predictive value, and negative predictive value were calculated at the Youden-optimal threshold. Internal validation was performed using 1000 bootstrap resamples, from which optimism-corrected AUCs were derived. Overall performance was summarized using the Brier score, and apparent calibration was assessed using the Hosmer-Lemeshow test with 10 risk groups. Clinical utility was evaluated using decision curve analysis. Because the endpoint was prevalent VCF status, all model analyses were interpreted as cross-sectional identification rather than prospective fracture prediction.

## 3. Results

### 3.1. Study Cohort and Baseline Characteristics

A total of 275 women with breast cancer were included in the final analysis. Baseline characteristics stratified by VCF status are summarized in Table 1. Moderate-to-severe VCF, defined as Genant grade ≥ 2, was present in 75 patients (27.3%), whereas 200 patients (72.7%) had Genant grade < 2. Patients with Genant grade ≥ 2 were older than those with Genant grade < 2 (58.9 ± 11.2 vs. 50.7 ± 9.1 years, *p* < 0.001) and had a slightly higher body mass index (23.3 ± 2.2 vs. 22.7 ± 2.2 kg/m^2^, *p* = 0.031). Adjuvant endocrine therapy was more frequent in the Genant grade ≥ 2 group (72.0% vs. 54.5%, *p* = 0.009), whereas radiotherapy did not differ significantly between groups (56.0% vs 46.5%, *p* = 0.160).

All bone density metrics differed significantly by VCF status. Patients with Genant grade ≥ 2 had lower QCT-vBMD than those with Genant grade < 2 (95.4 ± 29.7 vs. 127.5 ± 39.3 mg/cm^3^, *p* < 0.001). Similar differences were observed for HU-vBMD (94.3 [78.4–118.6] vs 122.1 [99.9–151.5] mg/cm^3^, *p* < 0.001) and AI-vBMD (92.6 [73.7–112.0] vs. 124.0 [98.4–150.8] mg/cm^3^, *p* < 0.001). The paraspinal IMAT ratio also differed significantly by VCF status. The median paraspinal IMAT ratio was higher in patients with Genant grade ≥ 2 than in those with Genant grade < 2 (0.076 [0.065–0.093] vs. 0.052 [0.040–0.070], *p* < 0.001), consistent with greater paraspinal intermuscular fat infiltration in the moderate-to-severe VCF group.

### 3.2. Agreement of Opportunistic CT-Derived Bone Density Metrics with QCT-vBMD

**Figure 2:**
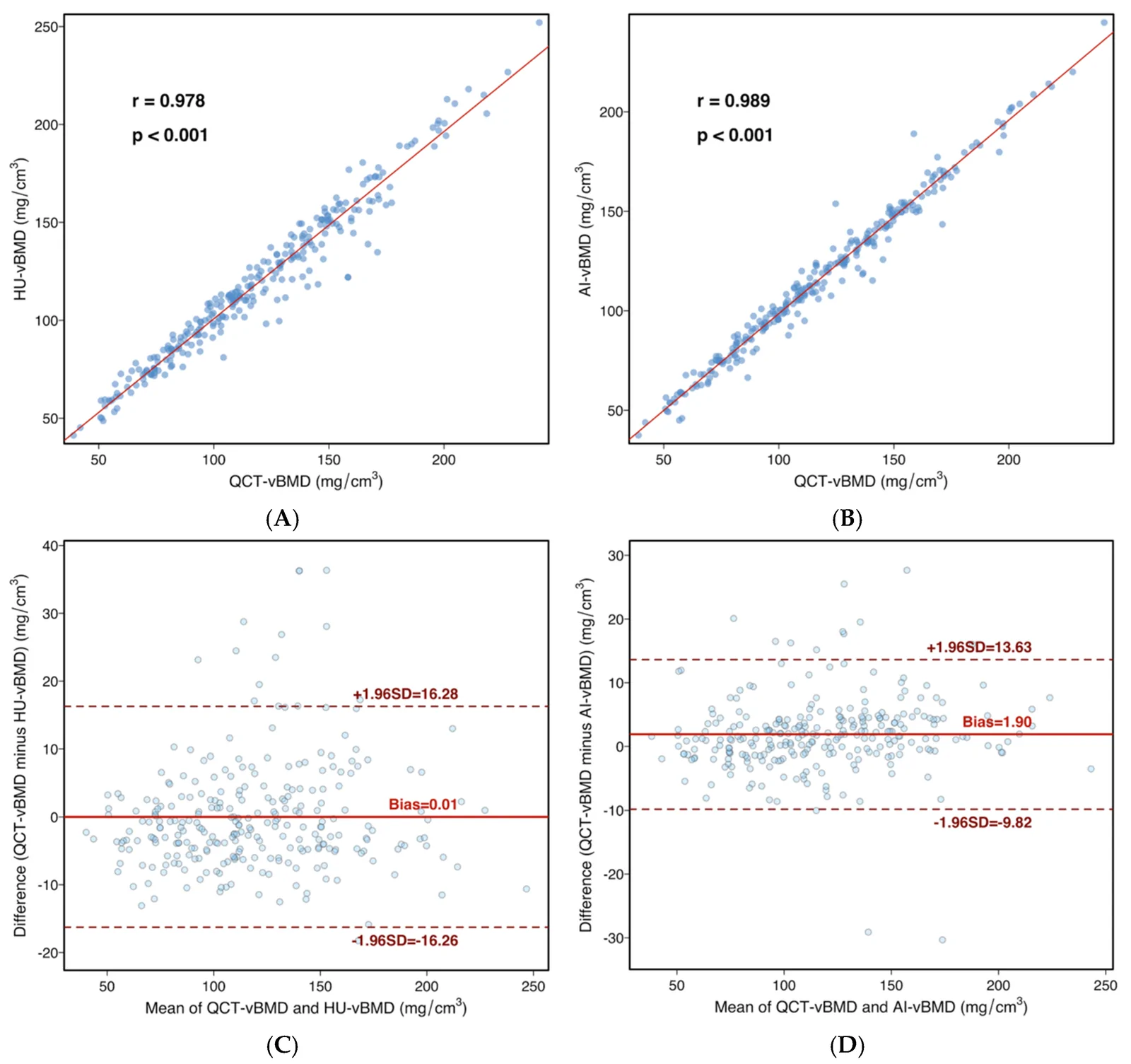
**Correlation and Bland–Altman analyses of CT-derived vBMD metrics against QCT-vBMD.** (**A**) Correlation between QCT-vBMD and HU-vBMD. (**B**) Correlation between QCT-vBMD and AI-vBMD. (**C**) Bland–Altman plot comparing QCT-vBMD and HU-vBMD. (**D**) Bland–Altman plot comparing QCT-vBMD and AI-vBMD. In panels (**A**,**B**), the red line represents the fitted linear regression line. In panels (**C**,**D**), the solid red line indicates the mean difference, and the dashed red lines indicate the 95% limits of agreement. Bland–Altman differences were calculated as QCT-vBMD minus the corresponding CT-derived vBMD metric. Abbreviations: AI-vBMD, artificial intelligence-derived volumetric bone mineral density; HU-vBMD, Hounsfield unit-derived volumetric bone mineral density; QCT-vBMD, quantitative CT-derived volumetric bone mineral density.

Both opportunistic CT-derived bone density metrics showed excellent agreement with the QCT reference standard (Table 2; Figure 2A–D). QCT-vBMD and HU-vBMD were very strongly correlated (Pearson r = 0.978, *p* < 0.001) and demonstrated excellent absolute agreement (ICC = 0.978, 95% CI 0.972–0.982). Because HU-vBMD was derived from a cohort-specific regression equation fitted against QCT-vBMD, this agreement should be interpreted as within-cohort calibration rather than independent validation. Similarly, QCT-vBMD and AI-vBMD showed an equally strong correlation (Pearson r = 0.989, *p* < 0.001) with excellent agreement (ICC = 0.987, 95% CI 0.984–0.990). HU-vBMD and AI-vBMD were also highly concordant with each other (Pearson r = 0.984, *p* < 0.001; ICC = 0.983, 95% CI 0.978–0.986).

Bland–Altman analysis further supported these findings. The mean difference between QCT-vBMD and HU-vBMD was 0.01 mg/cm^3^, with 95% limits of agreement from −16.26 to 16.28 mg/cm^3^. For QCT-vBMD versus AI-vBMD, the mean difference was 1.90 mg/cm^3^, with narrower 95% limits of agreement from −9.82 to 13.63 mg/cm^3^, indicating slightly closer agreement of AI-vBMD with the QCT reference. To express HU-derived attenuation on the QCT-referenced vBMD scale, HU-vBMD was derived from the cohort-specific linear regression of QCT-vBMD on mean L1–L3 HU values. The resulting conversion equation was: QCT-vBMD (mg/cm^3^) = 0.7923 × HU + 6.9283 (R^2^ = 0.956).

### 3.3. Reliability of the Imaging Endpoint

Inter-reader agreement for Genant semiquantitative grading was excellent on the initial independent reads (quadratic-weighted Cohen’s κ = 0.824; 95% CI, 0.789–0.854). Agreement for the binary endpoint of moderate-to-severe vertebral compression fracture (Genant grade ≥ 2) was also high (Cohen’s κ = 0.800; 95% CI, 0.713–0.875), with 92.0% exact agreement, supporting the robustness of the primary imaging endpoint.

### 3.4. Incremental Value of Prespecified Models for Detecting Moderate-to-Severe VCF

**Figure 3:**
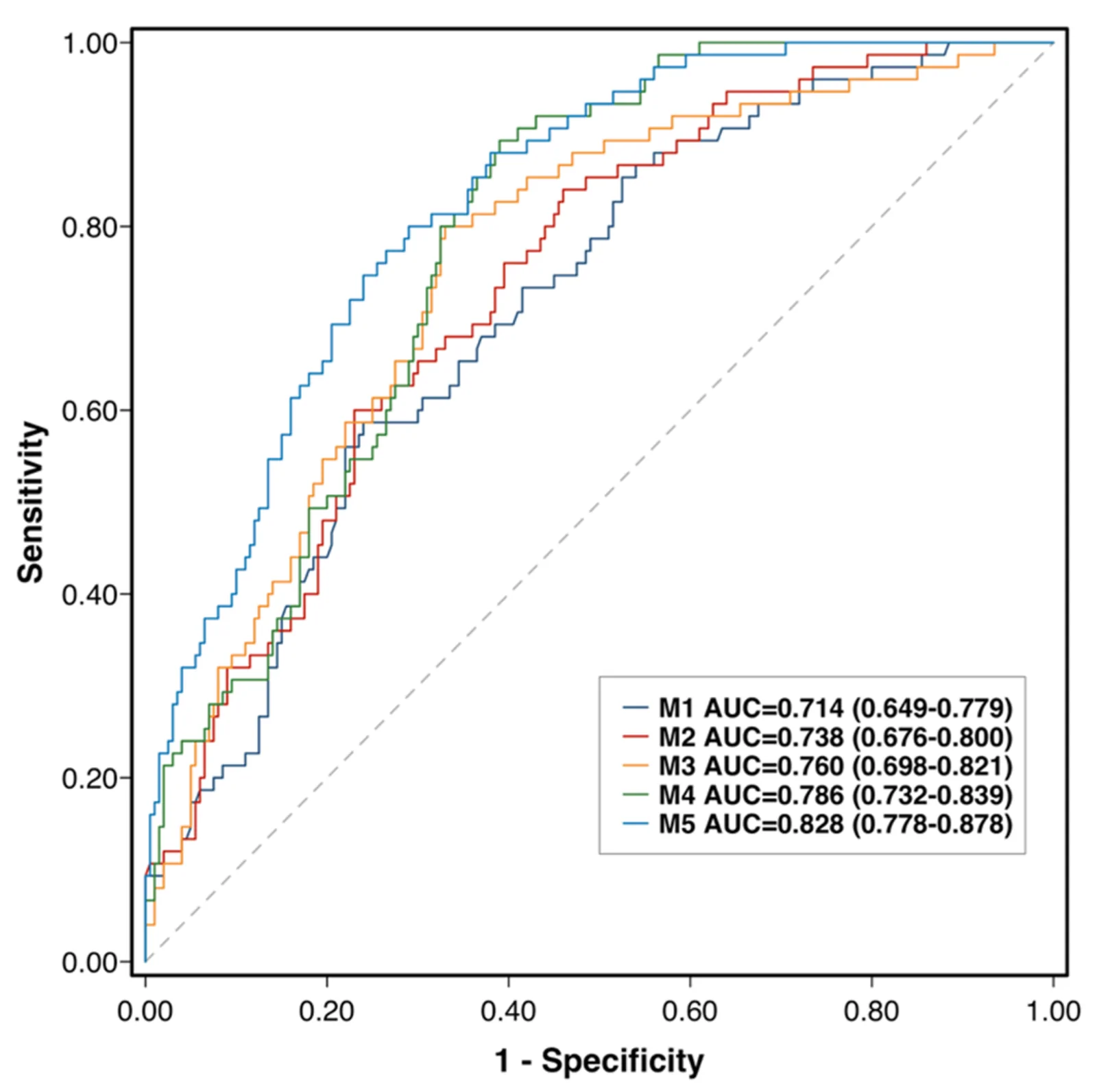
**ROC curves of prespecified models for identifying moderate-to-severe VCF.** Receiver operating characteristic curves are shown for the HU-vBMD-only model (M1), AI-vBMD-only model (M2), paraspinal IMAT ratio-only model (M3), AI-vBMD plus IMAT ratio model (M4), and the full imaging–clinical model (M5). Moderate-to-severe vertebral compression fracture was defined as Genant grade ≥ 2. Areas under the curve and 95% confidence intervals are reported in Table 4. Abbreviations: AI-vBMD, artificial intelligence-derived volumetric bone mineral density; AUC, area under the curve; HU-vBMD, Hounsfield unit-derived volumetric bone mineral density; IMAT, intermuscular adipose tissue; VCF, vertebral compression fracture.

All prespecified models were fitted in the full cohort (*n* = 275; events = 75). Logistic regression results are summarized in Table 3, and overall model performance is shown in Table 4 and Figure 3. Discrimination increased from single-marker models to combined imaging and imaging–clinical models. The HU-vBMD-only model (M1) yielded an AUC of 0.714 (95% CI, 0.649–0.779). The AI-vBMD-only model (M2) showed significantly better discrimination than M1, with an AUC of 0.738 (95% CI, 0.676–0.800; paired DeLong *p* < 0.001). The IMAT ratio-only model (M3) achieved an AUC of 0.760 (95% CI, 0.698–0.821), which was numerically higher than that of M2 but not significantly different (*p* = 0.604).

**Figure 4:**
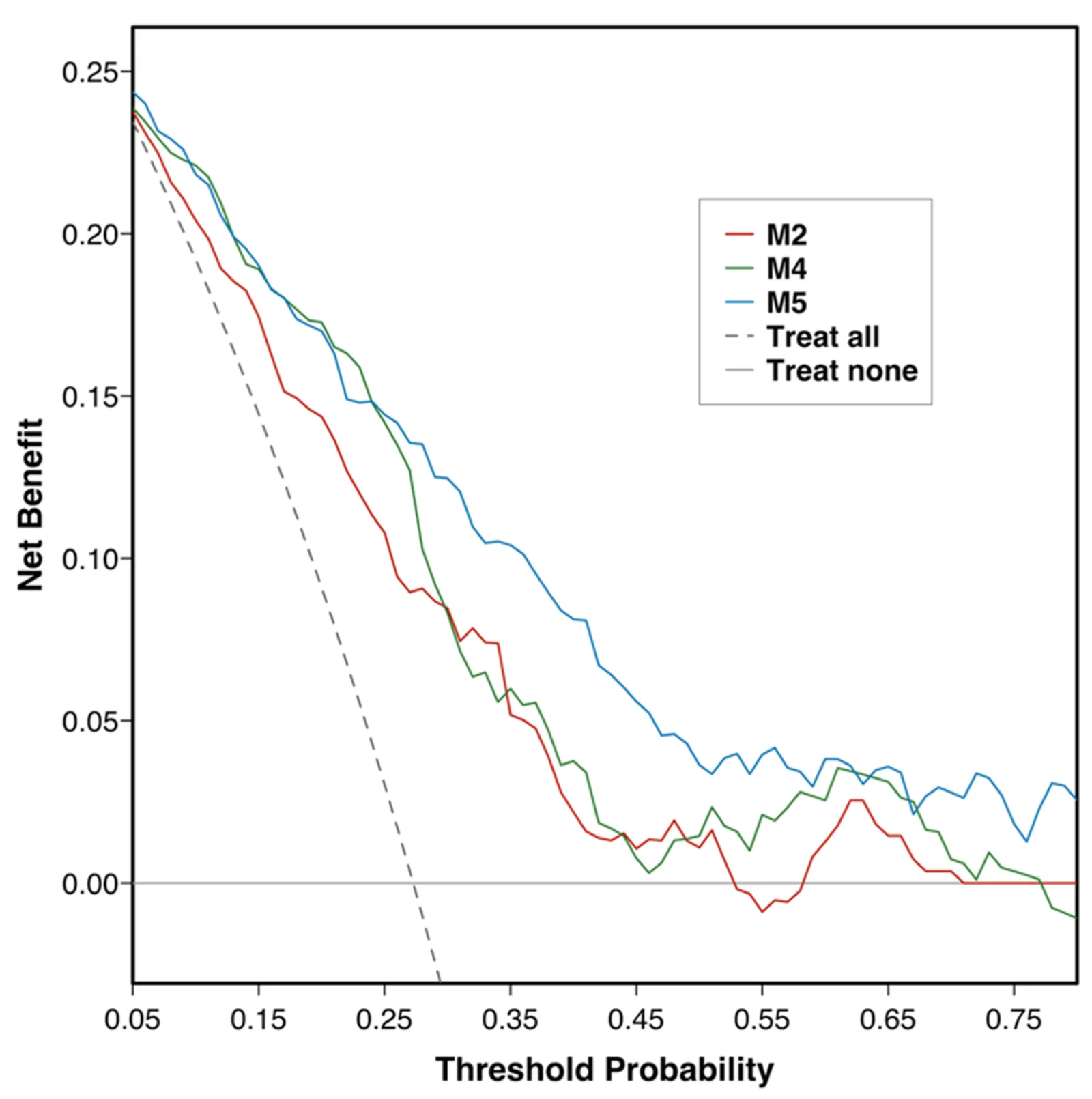
**DCA of selected models for identifying moderate-to-severe vertebral compression fractures.** Decision curve analysis compared the clinical net benefit of AI-vBMD alone (M2), AI-vBMD plus paraspinal IMAT ratio (M4), and the full imaging–clinical model (M5) across a range of threshold probabilities. The treat-all and treat-none strategies are shown as reference curves. Moderate-to-severe vertebral compression fracture was defined as Genant grade ≥ 2. Abbreviations: AI-vBMD, artificial intelligence-derived volumetric bone mineral density; IMAT, intermuscular adipose tissue; VCF, vertebral compression fracture.

Combining AI-vBMD with IMAT ratio (M4) further improved discrimination to an AUC of 0.786 (95% CI, 0.732–0.839), representing a significant improvement over AI-vBMD alone (M2 vs. M4, *p* = 0.038). The full model incorporating AI-vBMD, IMAT ratio, age, body mass index, adjuvant endocrine therapy, and radiotherapy (M5) achieved the highest discrimination, with an AUC of 0.828 (95% CI, 0.778–0.878), and significantly outperformed M4 (*p* = 0.035). Overall model performance also improved in parallel, with Brier scores decreasing from 0.1761 in M1 to 0.1452 in M5. In multivariable analyses, both AI-vBMD and IMAT ratio remained independently associated with the presence of moderate-to-severe VCF in the combined models. In M4, AI-vBMD was inversely associated with VCF (OR, 0.47; 95% CI, 0.32–0.67; *p* < 0.001), whereas IMAT ratio was positively associated with VCF (OR, 2.10; 95% CI, 1.50–3.03; *p* < 0.001). In the fully adjusted model (M5), AI-vBMD remained independently associated with lower odds of VCF (OR, 0.59; 95% CI, 0.35–0.99; *p* = 0.048), and IMAT ratio remained independently associated with higher odds of VCF (OR, 2.32; 95% CI, 1.62–3.44; *p* < 0.001). Among the clinical covariates, adjuvant endocrine therapy was independently associated with VCF status (OR, 4.10; 95% CI, 2.04–8.70; *p* < 0.001), whereas age, body mass index, and radiotherapy were not statistically significant in the fully adjusted model. Decision curve analysis showed that M5 provided the greatest net benefit among the evaluated models across most clinically relevant threshold probabilities, with M4 generally outperforming AI-vBMD alone (Figure 4). Youden-optimal threshold values are provided in Supplementary Table S2, and bootstrap internal validation results are summarized in Supplementary Table S3. The overall ranking of model discrimination was preserved after optimism correction, and M4 showed evidence of apparent miscalibration (HL *p* = 0.027).

## 4. Discussion

In this retrospective cohort study of 275 breast cancer survivors, we evaluated an automated CT-based approach for opportunistic extraction of AI-vBMD and paraspinal IMAT ratio from routine non-contrast CT. Three findings merit emphasis. First, both HU-vBMD and AI-vBMD showed excellent agreement with QCT-vBMD. Second, the paraspinal IMAT ratio was independently associated with prevalent moderate-to-severe VCFs. Third, adding the IMAT ratio to AI-vBMD improved fracture discrimination, and the full model incorporating clinical covariates achieved the highest overall performance. Together, these findings suggest that routine oncologic CT can be repurposed as a quantitative platform for integrated vertebral bone density and paraspinal muscle–fat assessment in breast cancer survivorship.

The agreement between CT-derived densitometry and QCT is a prerequisite for opportunistic bone assessment. QCT provides volumetric trabecular assessment and remains an established reference technique for advanced osteoporosis evaluation [8]. In the present study, both regression-derived HU-vBMD and AI-vBMD were closely aligned with QCT-vBMD, supporting the feasibility of extracting quantitative skeletal information from routine non-contrast oncologic CT. This finding is consistent with prior work showing that vertebral attenuation and CT-derived BMD can provide value-added skeletal information from examinations obtained for other indications [10,11,22]. The statistically significant but modest improvement of AI-vBMD over HU-vBMD should be interpreted in terms of both performance and workflow. HU-vBMD performed reasonably well, suggesting that a transparent QCT-referenced HU conversion remains a practical low-barrier strategy when automated tools are unavailable. By comparison, the automated analysis workflow can derive both AI-vBMD and paraspinal IMAT ratio from the same routine CT examination, which may improve standardization and scalability for opportunistic skeletal assessment [12,13,14,20]. This advantage is particularly relevant in breast cancer surveillance, where CT examinations are frequently acquired for oncologic purposes but skeletal information is often not systematically used.

The performance of paraspinal IMAT ratio, averaged across L1–L3 and distinct from bone marrow adiposity or muscle attenuation-based myosteatosis, is especially important in this cohort. Although IMAT ratio was not statistically superior to AI-vBMD as a single marker, it yielded the highest numerical single-marker AUC, suggesting that paraspinal muscle–fat infiltration captures information not reducible to bone density. This may be partly explained by the nature of the endpoint. Moderate-to-severe VCF is a morphologic manifestation of accumulated structural compromise, not a densitometric diagnosis alone. Increased paraspinal IMAT may reflect impaired muscle quality, reduced musculoskeletal reserve, and diminished spinal support. Previous studies have linked paraspinal muscle degeneration and fatty infiltration to osteoporotic vertebral fracture occurrence or recurrence [15,17], and CT-based modeling studies have suggested that vertebral bone and paravertebral muscle features provide complementary information for fracture assessment [23]. In this context, our findings extend prior bone density-focused or general-population opportunistic CT workflows by showing that AI-vBMD and paraspinal IMAT ratio obtained from the same routine non-contrast examination may provide complementary information for identifying clinically relevant vertebral fragility in breast cancer survivorship [21,24].

This disease-specific context is central to the interpretation of our findings. Breast cancer survivors may be exposed to overlapping skeletal and body-composition stressors, including aging, menopause, endocrine therapy, adiposity, and treatment-related changes. Aromatase inhibitor therapy is well established as a driver of estrogen depletion, bone loss, and fracture risk in hormone-sensitive breast cancer [3,4]. In women with breast cancer starting aromatase inhibitor therapy, vertebral fractures are common, and fracture risk may not be fully captured by bone density alone [3,25]. Emerging evidence also suggests that aromatase inhibitor treatment may be associated with unfavorable body-composition changes, including increased fat mass and reduced lean mass over time [26]. In women undergoing aromatase inhibitor therapy, fat body mass, lean mass–fat mass interaction, and fat body mass progression have been associated with morphometric vertebral fractures or fracture progression [27,28]. These observations support the biological plausibility of the relatively strong performance of IMAT ratio in the present cohort. However, direct evidence linking endocrine therapy to paraspinal IMAT accumulation remains limited. IMAT ratio should therefore be interpreted as an integrated imaging marker of musculoskeletal vulnerability rather than as a treatment-specific biomarker.

The incremental value of combining AI-vBMD and IMAT ratio supports the interpretation that vertebral bone density and paraspinal muscle–fat infiltration represent partially distinct components of vertebral vulnerability. AI-vBMD reflects trabecular bone status, whereas IMAT ratio reflects the paraspinal muscle–fat compartment, which is biomechanically coupled to spinal loading and postural stability [16,29]. The significant AUC improvement after adding IMAT ratio to AI-vBMD indicates that a combined CT-derived phenotype may better represent vertebral fragility than bone density alone. In breast cancer survivorship, this distinction is clinically relevant because the aim is not only to identify low BMD, but also to recognize patients with unreported structural compromise and broader musculoskeletal vulnerability.

The improvement observed after adding clinical covariates further suggests that imaging phenotypes and clinical context provide related but non-identical information. In breast cancer care, age, body mass index, radiotherapy, and adjuvant endocrine therapy are routinely available and clinically meaningful. The full model achieved the highest AUC and lowest Brier score, with more favorable apparent calibration than the imaging-only model, suggesting that clinical context may help align imaging-derived information with observed fracture status. M4 showed evidence of apparent calibration misfit despite improved discrimination, whereas M5 demonstrated the most favorable combined discrimination and apparent calibration profile. Among the clinical covariates, adjuvant endocrine therapy was independently associated with prevalent VCF. This association is clinically plausible because aromatase inhibitor-based endocrine therapy is associated with accelerated bone loss and increased fracture risk [3,4], whereas tamoxifen may preserve bone mineral density in some postmenopausal women [30,31]. The positive association observed in this cohort may therefore reflect the overall skeletal impact of the endocrine regimens represented in the study population rather than the effect of a single therapeutic agent. Consequently, despite being modeled as a binary variable, endocrine therapy still provided clinically relevant information beyond imaging alone and likely contributed to the improved performance of M5. Its contribution should therefore be interpreted as reflecting overall endocrine treatment exposure rather than the effect of any specific regimen. In this oncologic cohort, vertebrae suspicious for metastatic involvement were excluded using predefined imaging criteria, and discrepant or equivocal cases were resolved by consensus. The attenuation of age in the fully adjusted model likely reflects shared prognostic information with CT-derived skeletal and muscle–fat phenotypes, particularly AI-vBMD, rather than evidence that age is clinically irrelevant.

From a clinical perspective, these findings align with contemporary cancer survivorship and aromatase inhibitor-associated bone loss management frameworks, which emphasize fracture-risk assessment, bone-density evaluation, and timely bone-directed or lifestyle interventions in patients receiving endocrine therapy [4,32,33]. Routine non-contrast oncologic CT could therefore serve not only for tumor surveillance but also as an imaging entry point for skeletal triage. Patients with low AI-vBMD, elevated paraspinal IMAT ratio, or unrecognized moderate-to-severe VCF may warrant vertebral fracture review, formal bone health assessment, endocrine therapy risk evaluation, and consideration of bone-directed or exercise-based interventions. This approach should not be viewed as a replacement for radiologist assessment of vertebral morphology or dedicated densitometry. Rather, it may provide a quantitative adjunct to help integrate bone health assessment into existing oncologic imaging workflows, particularly because VCFs are often asymptomatic and underreported on routine CT examinations [5,6]. In practice, automated extraction could be embedded into routine oncology imaging pipelines as a background quantitative workflow that flags low bone density, elevated paraspinal IMAT, or possible unrecognized vertebral fracture for targeted clinical review. Wider clinical adoption will still depend on workflow integration, external validation, and platform interoperability.

Several limitations should be acknowledged. First, this was a retrospective single-center study, and the requirement for both true non-contrast CT and QCT within 7 days may also have selected a more highly characterized clinical subgroup, which may limit generalizability to broader breast cancer imaging populations. Second, the endpoint was prevalent rather than incident VCF; therefore, the models should be interpreted as tools for cross-sectional identification rather than prospective fracture prediction. Third, clinical treatment and patient-level factors were incompletely characterized. Detailed information on endocrine therapy regimen, duration, adherence, cumulative exposure, antiresorptive treatment, menopausal status, chemotherapy exposure, and lifestyle-related factors was limited, which may have introduced residual confounding, particularly for treatment-related associations. Fourth, quantitative bone density and paraspinal muscle–fat metrics were derived from L1–L3 rather than the full thoracolumbar spine, which may limit anatomic generalizability. Fifth, several technical and modeling limitations should be considered. HU-vBMD was derived from a cohort-specific regression equation fitted against QCT-vBMD and therefore reflects within-cohort calibration rather than independent validation. The findings for AI-vBMD and IMAT ratio are specific to the software version used; moreover, the detailed attenuation thresholds and proprietary segmentation rules of the commercial software were not accessible to the investigators, which limits methodological transparency and independent reproducibility of the IMAT-related measurements. Broader validation across CT vendors, reconstruction settings, scanner platforms, software implementations, and patient populations is therefore needed. Although the events-per-variable ratio and bootstrap validation supported acceptable internal stability, estimates for individual clinical covariates should be interpreted cautiously, and the M4 showed apparent calibration misfit despite preserved discrimination after bootstrap validation.

## 5. Conclusions

CT-derived HU-vBMD and AI-vBMD showed excellent agreement with QCT-vBMD in women with breast cancer. AI-derived paraspinal IMAT ratio provided complementary information to AI-vBMD for identifying Genant grade ≥2 VCF, and the combined imaging–clinical model achieved the highest discrimination. These findings support the potential role of routine non-contrast CT as a platform for integrated vertebral bone density and paraspinal muscle–fat assessment and opportunistic identification of clinically relevant vertebral fragility in breast cancer survivors.

## Figures and Tables

**Table 1:** **Baseline Characteristics Stratified by Vertebral Compression** **Fracture Status.**

Characteristic	Overall (*n* = 275)	Genant < 2 (*n* = 200)	Genant ≥ 2 (*n* = 75)	*p* Value
**Clinical characteristics**				
Age (years)	52.9 ± 10.4	50.7 ± 9.1	58.9 ± 11.2	<0.001
BMI (kg/m^2^)	22.9 ± 2.2	22.7 ± 2.2	23.3 ± 2.2	0.031
Adjuvant endocrine therapy, *n* (%)	163 (59.3%)	109 (54.5%)	54 (72.0%)	0.009
Radiotherapy, *n* (%)	135 (49.1%)	93 (46.5%)	42 (56.0%)	0.160
**Bone mineral density**				
QCT-vBMD (mg/cm^3^)	118.7 ± 39.6	127.5 ± 39.3	95.4 ± 29.7	<0.001
HU-vBMD (mg/cm^3^)	113.1 (89.7–144.4)	122.1 (99.9–151.5)	94.3 (78.4–118.6)	<0.001
AI-vBMD (mg/cm^3^)	112.5 (87.8–143.5)	124.0 (98.4–150.8)	92.6 (73.7–112.0)	<0.001
**Paraspinal muscle and fat phenotype**				
IMAT ratio	0.060 (0.043–0.079)	0.052 (0.040–0.070)	0.076 (0.065–0.093)	<0.001

Abbreviations: BMI, body mass index; QCT-vBMD, quantitative CT-derived volumetric bone mineral density; HU-vBMD, Hounsfield unit-derived volumetric bone mineral density; AI-vBMD, AI-derived volumetric bone mineral density; IMAT, intermuscular adipose tissue. Continuous variables are presented as mean ± SD for normally distributed data or median (IQR) for non-normally distributed data according to the Shapiro–Wilk normality test. Categorical variables are presented as *n* (%). Two-sided *p* values are reported. Among the 275 patients in the final complete case cohort, no missing data were present for the variables analyzed.

**Table 2:** **Correlation and Agreement Between CT-Derived BMD Metrics and** **the QCT Reference Standard.**

Comparison	*n*	Pearson r (95% CI)	*p* Value	ICC (95% CI)	BA Mean Diff. (mg/cm^3^)	95% LoA (mg/cm^3^)
QCT-vBMD vs. HU-vBMD	275	0.978 (0.969–0.985)	<0.001	0.978 (0.972–0.982)	0.01	−16.26 to 16.28
QCT-vBMD vs. AI-vBMD	275	0.989 (0.983–0.993)	<0.001	0.987 (0.984–0.990)	1.90	−9.82 to 13.63
HU-vBMD vs. AI-vBMD	275	0.984 (0.978–0.989)	<0.001	0.983 (0.978–0.986)	1.90	−11.77 to 15.57

Pearson correlation coefficients are presented with 95% confidence intervals. ICC denotes the intraclass correlation coefficient (two-way random-effects model, absolute agreement, single measurement). Bland–Altman (BA) mean difference was calculated as the first metric minus the second metric in each comparison. HU-vBMD was derived from the regression equation: QCT-vBMD = 0.7923 × raw HU + 6.9283 (R^2^ = 0.956); therefore, its agreement with QCT-vBMD reflects within-cohort calibration rather than external validation.

**Table 3:** **Logistic Regression Models for** **Identifying Moderate-to-Severe Vertebral Compression Fracture (Genant ≥ 2).**

Model/Predictor	Odds Ratio	95% CI	*p* Value	AUC (95% CI)	*n*
**M1: HU-vBMD**				0.714 (0.649–0.779)	275
HU-vBMD (per 1 SD)	0.40	0.27–0.55	<0.001		
**M2: AI-vBMD**				0.738 (0.676–0.800)	275
AI-vBMD (per 1 SD)	0.36	0.25–0.50	<0.001		
**M3: IMAT ratio**				0.760 (0.698–0.821)	275
IMAT ratio (per 1 SD)	2.62	1.92–3.68	<0.001		
**M4: M2 + IMAT ratio**				0.786 (0.732–0.839)	275
AI-vBMD (per 1 SD)	0.47	0.32–0.67	<0.001		
IMAT ratio (per 1 SD)	2.10	1.50–3.03	<0.001		
**M5: M4 + clinical covariates**				0.828 (0.778–0.878)	275
AI-vBMD (per 1 SD)	0.59	0.35–0.99	0.048		
IMAT ratio (per 1 SD)	2.32	1.62–3.44	<0.001		
Age (per 1 SD)	1.46	0.93–2.31	0.104		
BMI (per 1 SD)	1.12	0.80–1.56	0.514		
Adjuvant endocrine therapy (yes)	4.10	2.04–8.70	<0.001		
Radiotherapy (yes)	1.87	0.97–3.66	0.063		

Continuous predictors were z-score standardized before model fitting. Odds ratios are expressed per 1-SD increase for continuous variables. All models were fitted using standard logistic regression. AUCs are presented with 95% confidence intervals derived from ROC analysis.

**Table 4:** **Overall Performance of Prespecified Models for Identifying Moderate-to-Severe** **VCFs.**

Model	*n*	AUC (95% CI)	Sensitivity	Specificity	PPV	NPV	Brier Score	HL *p*	Key DeLong Comparison
M1	275	0.714 (0.649–0.779)	0.587	0.760	0.478	0.831	0.1761	0.946	M1 vs M2: <0.001; M1 vs M3: 0.269
M2	275	0.738 (0.676–0.800)	0.840	0.540	0.406	0.900	0.1718	0.886	M2 vs M3: 0.604; M2 vs M4: 0.038
M3	275	0.760 (0.698–0.821)	0.800	0.670	0.476	0.899	0.1684	0.595	M1 vs M3: 0.269; M2 vs M3: 0.604
M4	275	0.786 (0.732–0.839)	0.893	0.610	0.462	0.938	0.1605	0.027	M2 vs M4: 0.038; M4 vs M5: 0.035
M5	275	0.828 (0.778–0.878)	0.800	0.710	0.508	0.904	0.1452	0.733	M4 vs M5: 0.035

Sensitivity, specificity, positive predictive value (PPV), and negative predictive value (NPV) were calculated at the Youden-optimal threshold. Brier score reflects overall probabilistic accuracy, with lower values indicating better performance. Hosmer–Lemeshow (HL) *p* values are reported as a calibration summary. Only prespecified key DeLong comparisons are shown in the main table; their complete one-comparison-per-row presentation is provided in Supplementary Table S1. Bootstrap internal validation results are summarized in Supplementary Table S3.

## Data Availability

The data underlying this study are not publicly available due to institutional and ethical restrictions related to patient privacy. De-identified data and analysis code may be available from the corresponding author upon reasonable request and subject to institutional approval.

## Supplementary Materials

The following supporting information can be downloaded at: https://www.mdpi.com/article/10.3390/jcm15166283/s1, Table S1: Prespecified Paired DeLong Comparisons Among the Five Models; Table S2: Data-Derived Youden-Optimal Classification Thresholds Used for Model Performance Evaluation; Table S3: Bootstrap Internal Validation Summary of the Prespecified Models; Table S4: Collinearity Diagnostics and Predictor Correlations for Model 5.

## Abbreviations

The following abbreviations are used in this manuscript:

AI	Artificial intelligence
AI-vBMD	Artificial intelligence-derived volumetric bone mineral density
AUC	Area under the curve
BMI	Body mass index
CI	Confidence interval
CT	Computed tomography
DCA	Decision curve analysis
HU	Hounsfield unit
HU-vBMD	Hounsfield unit-derived volumetric bone mineral density
ICC	Intraclass correlation coefficient
IMAT	Intermuscular adipose tissue
NPV	Negative predictive value
PPV	Positive predictive value
QCT	Quantitative computed tomography
ROC	Receiver operating characteristic
VCF	Vertebral compression fracture
vBMD	Volumetric bone mineral density

## References

[1] Runowicz C.D., Leach C.R., Henry N.L., Henry K.S., Mackey H.T., Cowens-Alvarado R.L., Cannady R.S., Pratt-Chapman M.L., Edge S.B., Jacobs L.A. (2016). American Cancer Society/American Society of Clinical Oncology Breast Cancer Survivorship Care Guideline. J. Clin. Oncol..

[2] Bray F., Laversanne M., Sung H., Ferlay J., Siegel R.L., Soerjomataram I., Jemal A. (2024). Global cancer statistics 2022: GLOBOCAN estimates of incidence and mortality worldwide for 36 cancers in 185 countries. CA Cancer J. Clin..

[3] Bouvard B., Hoppé E., Soulié P., Georgin-Mege M., Jadaud E., Abadie-Lacourtoisie S., Petit Le Manac’h A., Laffitte A., Levasseur R., Audran M. (2012). High prevalence of vertebral fractures in women with breast cancer starting aromatase inhibitor therapy. Ann. Oncol..

[4] Hadji P., Aapro M., Al-Dagri N., Alokail M., Biver E., Body J.J., Brandi M.L., Brown J., Confavreux C., Cortet B. (2025). Management of aromatase inhibitor-associated bone loss (AIBL) in women with hormone-sensitive breast cancer: An updated joint position statement of the IOF, CABS, ECTS, IEG, ESCEO, IMS, and SIOG. J. Bone Oncol..

[5] Williams A.L., Al-Busaidi A., Sparrow P.J., Adams J.E., Whitehouse R.W. (2009). Under-reporting of osteoporotic vertebral fractures on computed tomography. Eur. J. Radiol..

[6] Woo E.K., Mansoubi H., Alyas F. (2008). Incidental vertebral fractures on multidetector CT images of the chest: Prevalence and recognition. Clin. Radiol..

[7] Genant H.K., Wu C.Y., van Kuijk C., Nevitt M.C. (1993). Vertebral fracture assessment using a semiquantitative technique. J. Bone Min. Res..

[8] Engelke K., Lang T., Khosla S., Qin L., Zysset P., Leslie W.D., Shepherd J.A., Shousboe J.T. (2015). Clinical Use of Quantitative Computed Tomography-Based Advanced Techniques in the Management of Osteoporosis in Adults: The 2015 ISCD Official Positions-Part III. J. Clin. Densitom..

[9] Boutin R.D., Lenchik L. (2020). Value-Added Opportunistic CT: Insights Into Osteoporosis and Sarcopenia. AJR Am. J. Roentgenol..

[10] Pickhardt P.J. (2022). Value-added Opportunistic CT Screening: State of the Art. Radiology.

[11] Pickhardt P.J., Pooler B.D., Lauder T., del Rio A.M., Bruce R.J., Binkley N. (2013). Opportunistic screening for osteoporosis using abdominal computed tomography scans obtained for other indications. Ann. Intern Med..

[12] Li W., Weng Y., Zong R., Wei M., Zheng C., Wu M., Zhou W., Pu J., Lu W., Lv F. (2025). Automatic phantom-less calibration of routine CT scans for the evaluation of osteoporosis and hip fracture risk. Bone.

[13] Yang J., Liao M., Wang Y., Chen L., He L., Ji Y., Xiao Y., Lu Y., Fan W., Nie Z. (2022). Opportunistic osteoporosis screening using chest CT with artificial intelligence. Osteoporos. Int..

[14] Westerhoff M., Gyftopoulos S., Dane B., Vega E., Murdock D., Lindow N., Herter F., Bousabarah K., Recht M.P., Bredella M.A. (2025). Deep Learning-based Opportunistic CT Osteoporosis Screening and the Establishment of Normative Values. Radiology.

[15] Mallio C.A., Volterrani C., Bernetti C., Stiffi M., Greco F., Beomonte Zobel B. (2024). Exploring the interplay between paraspinal muscular status and bone health in osteoporosis and fracture risk: A comprehensive literature review on computed tomography (CT) and magnetic resonance imaging (MRI) studies. Quant. Imaging Med. Surg..

[16] Frost H.M. (2003). Bone’s mechanostat: A 2003 update. Anat. Rec. A Discov. Mol. Cell Evol. Biol..

[17] Chen Z., Shi T., Li W., Sun J., Yao Z., Liu W. (2022). Role of paraspinal muscle degeneration in the occurrence and recurrence of osteoporotic vertebral fracture: A meta-analysis. Front. Endocrinol..

[18] Jäckle K., Lüken S., Roch P.J., Klockner F.S., Reinhold M., Meier M.P., Hawellek T., Lehmann W., Weiser L. (2023). Effect of a Contrast Agent on Bone Mineral Density Measurement in the Spine and Hip Using QCT-Conversion Factor Recommendation. J. Clin. Med..

[19] Kutleša Z., Jerković K., Ordulj I., Budimir Mršić D. (2023). The effect of contrast media on CT measures of bone mineral density: A systematic review. Skelet. Radiol..

[20] Liu Z.J., Zhang C., Ma C., Qi H., Yang Z.H., Wu H.Y., Yang K.D., Lin J.Y., Wong T.M., Li Z.Y. (2022). Automatic phantom-less QCT system with high precision of BMD measurement for osteoporosis screening: Technique optimisation and clinical validation. J. Orthop. Transl..

[21] Pu J., Zhou W., Wei M., Li W., Xiao Y., Xie J., Lv F. (2026). Opportunistic Osteoporosis Screening in Breast Cancer Using AI-Derived Vertebral BMD from Routine CT: Validation Against QCT and Multivariable Diagnostic Modeling. J. Clin. Med..

[22] Jang S., Graffy P.M., Ziemlewicz T.J., Lee S.J., Summers R.M., Pickhardt P.J. (2019). Opportunistic Osteoporosis Screening at Routine Abdominal and Thoracic CT: Normative L1 Trabecular Attenuation Values in More than 20 000 Adults. Radiology.

[23] Kong S.H., Cho W., Park S.B., Choo J., Kim J.H., Kim S.W., Shin C.S. (2024). A Computed Tomography-Based Fracture Prediction Model With Images of Vertebral Bones and Muscles by Employing Deep Learning: Development and Validation Study. J. Med. Internet Res..

[24] Park H., Kang W.Y., Woo O.H., Lee J., Yang Z., Oh S. (2024). Automated deep learning-based bone mineral density assessment for opportunistic osteoporosis screening using various CT protocols with multi-vendor scanners. Sci. Rep..

[25] Pedersini R., Monteverdi S., Mazziotti G., Amoroso V., Roca E., Maffezzoni F., Vassalli L., Rodella F., Formenti A.M., Frara S. (2017). Morphometric vertebral fractures in breast cancer patients treated with adjuvant aromatase inhibitor therapy: A cross-sectional study. Bone.

[26] Pedersini R., Schivardi G., Laini L., Zamparini M., Bonalumi A., di Mauro P., Bosio S., Amoroso V., Villa N., Alberti A. (2024). Changes in body composition in early breast cancer patients treated with aromatase inhibitors. J. Endocrinol. Investig..

[27] Pedersini R., Amoroso V., Maffezzoni F., Gallo F., Turla A., Monteverdi S., Ardine M., Ravanelli M., Vassalli L., Rodella F. (2019). Association of Fat Body Mass With Vertebral Fractures in Postmenopausal Women With Early Breast Cancer Undergoing Adjuvant Aromatase Inhibitor Therapy. JAMA Netw. Open.

[28] Monteverdi S., Pedersini R., Gallo F., Maffezzoni F., Dalla Volta A., Di Mauro P., Turla A., Vassalli L., Ardine M., Formenti A.M. (2021). The Interaction of Lean Body Mass With Fat Body Mass Is Associated With Vertebral Fracture Prevalence in Women With Early Breast Cancer Undergoing Aromatase Inhibitor Therapy. JBMR Plus.

[29] Ma H.T., Griffith J.F., Xu L., Leung P.C. (2014). The functional muscle-bone unit in subjects of varying BMD. Osteoporos. Int..

[30] Love R.R., Mazess R.B., Barden H.S., Epstein S., Newcomb P.A., Jordan V.C., Carbone P.P., Demets D.L. (1992). Effects of tamoxifen on bone mineral density in postmenopausal women with breast cancer. N. Engl. J. Med..

[31] Bradley R., Braybrooke J., Gray R., Hills R.K., Liu Z., Pan H., Peto R., Dodwell D., Mcgale P., Taylor C. (2022). Aromatase inhibitors versus tamoxifen in premenopausal women with oestrogen receptor-positive early-stage breast cancer treated with ovarian suppression: A patient-level meta-analysis of 7030 women from four randomised trials. Lancet Oncol..

[32] Shapiro C.L., Van Poznak C., Lacchetti C., Kirshner J., Eastell R., Gagel R., Smith S., Edwards B.J., Frank E., Lyman G.H. (2019). Management of Osteoporosis in Survivors of Adult Cancers With Nonmetastatic Disease: ASCO Clinical Practice Guideline. J. Clin. Oncol..

[33] Campbell K.L., Winters-Stone K.M., Wiskemann J., May A.M., Schwartz A.L., Courneya K.S., Zucker D.S., Matthews C.E., Ligibel J.A., Gerber L.H. (2019). Exercise Guidelines for Cancer Survivors: Consensus Statement from International Multidisciplinary Roundtable. Med. Sci. Sports Exerc..

