# Supplementary material for: Beyond Bone Density Alone: Opportunistic Identification of Vertebral Compression Fractures in Breast Cancer Survivors Using Artificial Intelligence-Derived Vertebral Bone Density and Paraspinal Muscle–Fat Metrics
Source: J Clin Med. 2026 Aug 13;15(16):6283. doi: 10.3390/jcm15166283 (PMC13513670; doi:10.3390/jcm15166283)
Supplement: Supplementary file 1 [file jcm-15-06283-s001.zip › STARD checklist.pdf]

The STARD reporting checklist

STARD Checklist for *"**Beyond Bone Density Alone: Opportunistic Identification of Vertebral Compression Fractures in Breast Cancer Survivors Using Artificial Intelligence-Derived Vertebral Bone Density and Paraspinal Muscle-Fat Metrics**"*

	Item Description	Location (or reason for not reporting)
**Title or abstract**		
1. Identification as a study of diagnostic accuracy	Identification as a study of diagnostic accuracy using at least one measure of accuracy, such as sensitivity, specificity, predictive values, or AUC.	Title; Abstract—Objectives, Materials and Methods, and Results, pp. 1–2. The study identifies diagnostic performance for moderate-to-severe VCFs and reports AUCs and model discrimination.
**Abstract**		
2. Abstract	Structured summary of study design, methods, results, and conclusions.	Abstract, pp. 1–2. Structured summary includes Objectives, Materials and Methods, Results, and Conclusions.
**Introduction**		
3. Background	Scientific and clinical background, including the intended use and clinical role of the index test.	Introduction, pp. 2–3. The background describes breast cancer survivorship, skeletal fragility, under-recognition of VCFs, routine CT as an opportunity for opportunistic skeletal assessment, and the intended diagnostic role of AI-vBMD and paraspinal IMAT ratio.
4. Objectives	Study objectives and hypotheses.	Introduction, final paragraph, p. 3. The study objective is to evaluate agreement of HU-vBMD and AI-vBMD with QCT-vBMD and determine the diagnostic performance and incremental value of CT-derived bone density and AI-derived paraspinal IMAT ratio for identifying Genant grade ≥2 VCFs.
**Methods**		
5. Study design	Whether data collection was planned before the index test and reference standard were performed or after.	Materials and Methods opening paragraph, p. 3; Abstract—Materials and Methods, p. 1. The study is described as retrospective and non-interventional, using de-identified data. The manuscript also states that STARD was applied to the diagnostic accuracy analyses where applicable.
Participants		
6. Eligibility criteria	Eligibility criteria.	Methods 2.1 Participants, pp. 3–4. Eligibility criteria include consecutive adult women with histologically confirmed breast cancer, routine non-contrast CT with evaluable L1–L3 coverage, lumbar

		QCT within 7 days, and exclusion criteria including vertebral metastasis or malignant infiltration, vertebral instrumentation or augmentation, non-diagnostic CT image quality, incomplete QCT, or missing key clinical data. The manuscript also clarifies that neither current treatment status nor metastatic disease outside the spine was an exclusion criterion.
7. Identifying eligible participants	On what basis potentially eligible participants were identified.	Methods 2.1 Participants, pp. 3–4; Figure 1, p. 4. Potentially eligible participants were identified from consecutive women with histologically confirmed breast cancer who underwent routine non-contrast CT and lumbar QCT within the specified interval. Figure 1 reports the screening and analytic cohort flow.
8. Setting, location, and dates	Where and when potentially eligible participants were identified.	Methods 2.1 Participants, p. 3. The study was conducted at a single tertiary referral center; eligible participants were identified between June 2021 and November 2024.
9. Consecutive, random or convenience series	Whether participants formed a consecutive, random, or convenience series.	Methods 2.1 Participants, p. 3. Participants formed a consecutive retrospective series of eligible adult women with breast cancer.
Test Methods		
10. Index test & Reference standard	10a. Details of index test.<br><br>10b. Details of reference standard.	Methods 2.4 HU measurement and HU-derived vBMD, p. 5; Methods 2.5 AI-derived vBMD and paraspinal muscle-fat metrics from routine CT, pp. 5–6; Methods 2.7 Statistical analysis, pp. 6–7. Index tests included HU-vBMD, AI-vBMD, paraspinal IMAT ratio, and prespecified logistic regression models using these metrics. AI-derived measurements were obtained directly from automated software output without manual ROI delineation or manual quantitative adjustment.<br><br>Methods 2.6 Vertebral compression fracture assessment, p. 6. The diagnostic endpoint/reference standard was moderate-to-severe VCF, defined as Genant grade ≥2, assessed on sagittal multiplanar reformatted CT images covering T4–L5 by two blinded musculoskeletal radiologists, with consensus adjudication. Fractures suspicious for metastatic involvement were excluded according to predefined criteria.
11. Reference standard rationale	Rationale for choosing the reference standard, if alternatives exist.	Introduction, p. 2; Methods 2.6, p. 6. Genant grade ≥2 was selected because moderate-to-severe VCFs reflect substantial vertebral structural compromise

		and served as a clinically meaningful imaging endpoint.
12. Index test and reference standard cut-offs or categories	12a. Definition of and rationale for test positivity cut-offs or result categories of the index test, distinguishing prespecified from exploratory.<br><br>12b. Definition of and rationale for test positivity cut-offs or result categories of the reference standard, distinguishing prespecified from exploratory.	Methods 2.7 Statistical analysis, p. 6; Table 4, p. 12–13. Models M1–M5 were prespecified. Sensitivity, specificity, PPV, and NPV were calculated at the Youden-optimal threshold. No fixed clinical cut-off was prespecified for AI-vBMD or IMAT ratio; model-based diagnostic performance was evaluated by ROC analysis.<br><br>Methods 2.7 Statistical analysis, p. 6; Table 4, p. 12–13. Models M1–M5 were prespecified. Sensitivity, specificity, PPV, and NPV were calculated at the Youden-optimal threshold. No fixed clinical cut-off was prespecified for AI-vBMD or IMAT ratio; model-based diagnostic performance was evaluated by ROC analysis.
13. Information available to performers or readers of the index test and reference standard assessors	13a. Whether clinical information and reference-standard results were available to performers or readers of the index test.<br><br>13b. Whether clinical information and index-test results were available to assessors of the reference standard.	Methods 2.4–2.5, pp. 5–6; Methods 2.7, pp. 6–7. HU-vBMD, AI-vBMD, and IMAT ratio were derived from CT measurements and automated software outputs. M5 incorporated routinely available clinical variables as prespecified covariates.<br><br>Methods 2.6 Vertebral compression fracture assessment, p. 6. Radiologists assessing VCFs were blinded to HU-vBMD, QCT-vBMD, AI-derived measurements, model outputs, and clinical information.
Analysis		
14. Analysis methods	Methods for estimating or comparing measures of diagnostic accuracy.	Methods 2.7 Statistical analysis, pp. 6–7. Diagnostic accuracy was estimated using ROC curves and AUCs with 95% CIs. Paired DeLong tests compared model AUCs. Sensitivity, specificity, PPV, and NPV were calculated at Youden-optimal thresholds. Brier score, Hosmer–Lemeshow test, bootstrap internal validation, collinearity diagnostics, and decision curve analysis were also used.
15. Indeterminate results	How indeterminate index-test or reference-standard results were handled.	Methods 2.1 Participants, pp. 3–4; Methods 2.6, p. 6; Figure 1, p. 4. Non-diagnostic CT image quality, incomplete QCT results, missing key clinical information, and cases unsuitable for reliable evaluation were excluded according to predefined criteria. Discrepant VCF grades were re-reviewed after a washout period and resolved by consensus.

16. Missing data	How missing data on the index test and reference standard were handled.	Methods 2.1 Participants, pp. 3–4; Methods 2.7 Statistical analysis, pp. 6–7; Table 1 footnote, p. 8. Analyses used complete-case data. Patients with incomplete QCT or missing key clinical data were excluded. Table 1 states that no missing data were present for variables included in the final complete-case cohort.
17. Variability	Analyses of variability in diagnostic accuracy, distinguishing prespecified from exploratory.	Results 3.3 Reliability of the imaging endpoint, p. 10; Results 3.4, pp. 10–13. Inter-reader agreement for Genant grading was reported using quadratic-weighted Cohen's κ and Cohen's κ for the binary Genant grade ≥2 endpoint. No subgroup variability analysis of diagnostic accuracy was performed beyond prespecified model comparisons.
18. Intended sample size	Intended sample size and how it was determined.	Methods 2.1 Participants, pp. 3–4; Figure 1, p. 4. No formal a priori sample-size calculation was performed. The study included all eligible consecutive patients available during the predefined study period after predefined exclusions, yielding a final analytic cohort of 275 women with 75 moderate-to-severe VCF events. This addition addresses the STARD sample-size item specifically noted by the reviewer.
**Results**		
Participants		
19. Participant flow diagram	Flow of participants, using a diagram.	F Figure 1, p. 4. The flow diagram reports 311 screened patients, 36 exclusions with reasons, final analytic cohort of 275 women, VCF status, index assessments, and analyses performed.
20. Baseline characteristics	Baseline demographic and clinical characteristics of participants.	Results 3.1 Study cohort and baseline characteristics, pp. 7–8; Table 1, p. 8. Baseline demographic, clinical, bone density, and IMAT ratio characteristics are reported by VCF status.
21a. Participants with and without the target condition	21a. Distribution of severity of disease in those with the target condition.<br><br>21b. Distribution of alternative diagnoses in those without the target condition	Results 3.1, pp. 7–8; Table 1, p. 8; Figure 1, p. 4. The target condition was moderate-to-severe VCF, present in 75 patients (27.3%). Clinical and imaging characteristics of the Genant grade ≥2 group are reported.<br><br>Results 3.1, pp. 7–8; Table 1, p. 8; Figure 1, p. 4. The comparison group comprised 200 patients (72.7%) with Genant grade <2. Alternative diagnoses were not the focus of this diagnostic accuracy study; suspicious metastatic fractures were excluded according to predefined criteria.

22. Time interval	Time interval and any clinical interventions between index test and reference standard.	Methods 2.1 Participants, p. 3. Routine non-contrast CT and lumbar QCT were required to be performed within 7 days. No study-related clinical interventions occurred because this was a retrospective, non-interventional analysis of existing clinical imaging data.
Test Results		
23. Index test and reference standard results	Cross tabulation of the index test results (or their distribution) by the results of the reference standard.	Results 3.1–3.4, pp. 7–13; Tables 1, 3, and 4; Figures 3–4. Distributions of index-test variables by VCF status are shown in Table 1. Logistic regression results and diagnostic performance are summarized in Tables 3–4 and Figures 3–4. Because the main index tests were continuous markers and model-based predicted probabilities rather than fixed binary tests, conventional 2×2 cross-tabulations were not the primary analytic format; sensitivity, specificity, PPV, and NPV were reported at Youden-optimal thresholds.
24. Estimates of accuracy	Estimates of diagnostic accuracy and their precision (such as 95% CIs).	Abstract—Results, pp. 1–2; Results 3.4, pp. 10–13; Table 4, pp. 12–13; Figure 3, pp. 11–12; Supplementary Tables S1–S3. AUCs with 95% CIs, sensitivity, specificity, PPV, NPV, paired DeLong comparisons, Brier scores, Hosmer–Lemeshow calibration summaries, decision curve analysis, Youden-optimal thresholds, and bootstrap internal validation results are reported. Table 3 provides model ORs and AUCs; Table 4 summarizes diagnostic performance.
25. Adverse events	Any adverse events from performing the index test or the reference standard.	Not applicable. This was a retrospective analysis of existing, de-identified routine CT and QCT data with no additional imaging, intervention, or patient contact; therefore, no study-related adverse events occurred.
**Discussion**		
26. Limitations	Study limitations, including sources of potential bias, statistical uncertainty and generalisability.	Discussion—limitations paragraph, pp. 15–16. Limitations include retrospective single-center design, potential selection of a more highly characterized subgroup due to the requirement for true non-contrast CT and lumbar QCT within 7 days, prevalent rather than incident VCF endpoint, incomplete treatment and patient-level factors, L1–L3 rather than full thoracolumbar quantification, cohort-specific HU-vBMD calibration, software-version specificity, inaccessible proprietary attenuation thresholds and segmentation rules, need for broader validation, cautious

		interpretation of individual clinical covariates, and apparent M4 calibration misfit.
27. Implications for Practice	Implications for practice, including the intended use and clinical role of the index test.	Discussion—clinical perspective paragraph, p. 15; Conclusions, p. 16. The manuscript discusses routine non-contrast oncologic CT as an imaging entry point for skeletal triage and a quantitative adjunct for identifying patients who may warrant vertebral fracture review and formal bone health assessment. The approach is framed as an adjunct rather than a replacement for radiologist assessment or dedicated densitometry.
**Other information**		
28. Registration	Registration number and name of registry.	Not applicable. This was a retrospective, non-interventional diagnostic accuracy study using existing de-identified imaging and clinical data; no clinical trial registration was required.
29. Protocol	Where the full study protocol can be accessed.	Not publicly available. The retrospective study protocol was approved by the Ethics Review Committee of the First Affiliated Hospital of Chongqing Medical University (approval No. CYFYY276-02).
30. Funding	Sources of funding and other support; role of funders.	Funding statement, p. 16. The study was supported by the National Key R&D Program of China (grant no. 2020YFA0714002) and the Master's Research Innovation Project of the First Clinical College of Chongqing Medical University (no. CYYY-SSCX202525). The funders had no role in study design, data collection, analysis, interpretation, writing, or publication decision.

This checklist was completed based on the submitted manuscript entitled "***Beyond Bone Density Alone: Opportunistic Identification of Vertebral Compression Fractures in Breast Cancer Survivors Using Artificial Intelligence-Derived Vertebral Bone Density and Paraspinal Muscle-Fat Metrics.***" Items marked as not applicable reflect the retrospective, non-interventional design of the study and the use of existing de-identified imaging and clinical data.
