# Supplementary material for: Beyond Bone Density Alone: Opportunistic Identification of Vertebral Compression Fractures in Breast Cancer Survivors Using Artificial Intelligence-Derived Vertebral Bone Density and Paraspinal Muscle–Fat Metrics
Source: J Clin Med. 2026 Aug 13;15(16):6283. doi: 10.3390/jcm15166283 (PMC13513670; doi:10.3390/jcm15166283)
Supplement: Supplementary file 1 [file jcm-15-06283-s001.zip › Supplementary Table.pdf]

Supplementary Table S1. Prespecified Paired DeLong Comparisons Among the Five Models

Comparison	ΔAUC	Z statistic	P value
M1 vs M2	0.024	3.770	<0.001
M1 vs M3	0.046	1.106	0.269
M2 vs M3	0.021	0.519	0.604
M2 vs M4	0.047	2.078	0.038
M4 vs M5	0.042	2.108	0.035

Footnote: Prespecified paired AUC comparisons were performed using the analytic DeLong method. ΔAUC is defined as the AUC of the second model minus that of the first model.

Supplementary Table S2. Data-Derived Youden-Optimal Classification Thresholds Used for Model Performance Evaluation

Model	Youden-optimal threshold	Interpretation
M1	0.332	HU-vBMD ≤ 99.4 mg/cm³
M2	0.210	AI-vBMD ≤ 121.7 mg/cm³
M3	0.246	IMAT ratio ≥ 0.063
M4	0.231	Predicted probability threshold
M5	0.248	Predicted probability threshold

Footnote: Thresholds were selected by maximizing the Youden index within the study cohort and are provided to support the reported sensitivity, specificity, PPV, and NPV. These thresholds are data-derived classification cutoffs rather than established clinical decision thresholds. For single-marker models, the corresponding cutoffs are additionally expressed on the original marker scale. Because HU-vBMD and AI-vBMD were generated using different derivation frameworks, their absolute threshold values should not be interpreted as directly interchangeable.

Supplementary Table S3. Bootstrap Internal Validation Summary of the Prespecified Models

Model	Apparent AUC	AUC optimism	Optimism-corrected AUC	Optimism-corrected calibration intercept	Optimism-corrected calibration slope	Corrected Brier score
M1	0.714	0.000	0.714	0.022	1.014	0.1787
M2	0.738	-0.002	0.740	0.020	1.023	0.1734
M3	0.760	0.000	0.760	0.020	1.016	0.1707
M4	0.786	0.005	0.780	-0.018	0.972	0.1645
M5	0.828	0.019	0.808	-0.069	0.896	0.1551

Footnote: Internal validation was performed using 1000 bootstrap resamples. Optimism was calculated as the mean difference between model performance in the bootstrap sample and performance of the same fitted model when applied to the original cohort. The optimism-corrected AUC was calculated as the apparent AUC minus the mean bootstrap optimism. Calibration intercept and slope are reported as supplementary measures of internal calibration. Negative optimism estimates close to zero should be interpreted as indicating essentially no detectable optimism rather than true performance gain.

Supplementary Table S4. Collinearity Diagnostics and Predictor Correlations for Model 5

Panel A. Collinearity Diagnostics for Model 5

Predictor	VIF	Tolerance
AI-vBMD	2.24	0.447
IMAT ratio	1.23	0.810
Age	2.09	0.479
BMI	1.07	0.933
Adjuvant endocrine therapy	1.08	0.925
Radiotherapy	1.09	0.916

Footnote: Variance inflation factor (VIF) and tolerance are reported to assess multicollinearity in Model 5. No predictor showed evidence of problematic multicollinearity.

Panel B. Predictor Correlation Matrix for Model 5

	AI-vBMD	IMAT ratio	Age	BMI	Endocrine therapy	Radiotherapy
AI-vBMD	1.000	-0.421	-0.715	-0.207	0.071	-0.135
IMAT ratio	-0.421	1.000	0.340	0.172	-0.069	0.024
Age	-0.715	0.340	1.000	0.221	-0.009	0.104
BMI	-0.207	0.172	0.221	1.000	-0.060	-0.019
Endocrine therapy	0.071	-0.069	-0.009	-0.060	1.000	-0.252
Radiotherapy	-0.135	0.024	0.104	-0.019	-0.252	1.000

Footnote: Correlations were calculated using Pearson coefficients. For continuous-binary variable pairs, the values correspond to point-biserial correlations; for binary-binary pairs, they correspond to phi coefficients under 0/1 coding. The strongest observed correlation was between age and AI-vBMD ($r = -0.715$), but overall diagnostic metrics did not support problematic multicollinearity.
