# Supplementary material for: Beyond Bone Density Alone: Opportunistic Identification of Vertebral Compression Fractures in Breast Cancer Survivors Using Artificial Intelligence-Derived Vertebral Bone Density and Paraspinal Muscle–Fat Metrics
Source: J Clin Med. 2026 Aug 13;15(16):6283. doi: 10.3390/jcm15166283 (PMC13513670; doi:10.3390/jcm15166283)
Supplement: Supplementary file 1 [file jcm-15-06283-s001.zip › STROBE checklist.pdf]

STROBE Statement — Checklist of items that should be included in reports of cross-sectional studies

Beyond Bone Density Alone: Opportunistic Identification of Vertebral Compression Fractures in Breast Cancer Survivors Using Artificial Intelligence-Derived Vertebral Bone Density and Paraspinal Muscle-Fat Metrics

Note: This checklist corresponds to the cross-sectional design. Items not applicable to cross-sectional studies are marked as "N/A".

Item No.	Recommendation (abridged from STROBE)	Where reported (section / figure / table)	Notes
1(a)	Indicate the study design with a commonly used term in the title or the abstract	Title and Abstract—Materials and Methods, pp. 1–2.	The title identifies the diagnostic target and CT-derived AI markers. The abstract states that this was a retrospective study.
1(b)	Provide an informative, balanced structured abstract	Abstract, pp. 1–2.	Structured abstract includes Objectives, Materials and Methods, Results, and Conclusions.
2	Explain scientific background and rationale	Introduction, pp. 2–3.	Background and rationale are described, including breast cancer survivorship, skeletal fragility, under-recognition of VCFs, routine CT as an opportunity for opportunistic assessment, and the rationale for AI-vBMD and paraspinal IMAT ratio.
3	State specific objectives, including prespecified hypotheses	Introduction, final paragraph, p. 3.	Objectives are stated: to evaluate agreement of HU-vBMD and AI-vBMD with QCT-vBMD and to determine the diagnostic performance and incremental value of CT-derived bone density and AI-derived paraspinal IMAT ratio for identifying Genant grade ≥2 VCFs.
4	Present key elements of study design early in the paper	Materials and Methods opening paragraph, p. 3.	The study is described as retrospective and non-interventional, using de-identified data, with ethics approval. The manuscript also clarifies that STROBE was applied to the observational cross-sectional components and STARD to the diagnostic accuracy analyses where applicable.
5	Describe the setting, locations, and relevant dates	Methods 2.1 Participants, p. 3.	Setting, date range, and CT–QCT interval are described: single tertiary referral center, June 2021 to November 2024, routine non-contrast CT and lumbar QCT within 7 days.
6(a)	Give eligibility criteria and sources/methods of	Methods 2.1 Participants, pp. 3–4; Figure 1, pp. 4.	Eligibility criteria include consecutive adult women with histologically confirmed breast cancer, routine non-contrast CT with evaluable L1–L3 coverage, and lumbar QCT

	selection of participants		within 7 days. Exclusion criteria include vertebral metastasis or other malignant infiltration, vertebral instrumentation or augmentation, non-diagnostic CT image quality, incomplete QCT, or missing key clinical data. The manuscript also clarifies that neither current treatment status nor metastatic disease outside the spine was an exclusion criterion.
6(b)	For matched studies, give matching criteria and number of exposed/unexposed	N/A	Not a matched study
7	Define outcomes, exposures, predictors, potential confounders, and effect modifiers; give diagnostic criteria	Methods 2.1 and 2.4–2.7, pp. 3–7.	Outcomes, predictors, and covariates are defined: Genant grade ≥2 VCF as the endpoint; HU-vBMD, AI-vBMD, and paraspinal IMAT ratio as imaging predictors; age, BMI, adjuvant endocrine therapy, and radiotherapy as clinical covariates; M1–M5 as prespecified models.
8	For each variable, give data sources and methods of assessment; describe comparability	Methods 2.1–2.7, pp. 3–7.	Data sources and assessment methods are reported: clinical variables from medical records; CT acquisition protocol; QCT reference standard; HU measurement protocol; automated AI-derived vBMD and IMAT ratio; blinded Genant VCF assessment; and statistical modeling. AI-derived measurements were obtained directly from automated software output without manual ROI delineation or manual quantitative adjustment.
9	Describe any efforts to address potential sources of bias	Methods 2.1, 2.2, 2.6, and 2.7, pp. 3–7.	Efforts include consecutive patient selection, CT–QCT interval ≤7 days, inclusion of only true non-contrast CT scans, standardized CT acquisition, exclusion of metastatic/instrumented/non-diagnostic cases, blinded dual-reader Genant grading, computer-generated random reading order, consensus adjudication, complete-case analysis, and bootstrap internal validation.
10	Explain how the study size was arrived at	Methods 2.1 Participants, pp. 3–4; Figure 1, p. 4; Results 3.1, pp. 7–8.	No formal a priori sample-size calculation was performed. The study included all consecutive eligible patients during the predefined study period after predefined exclusions, yielding a final cohort of 275 women with 75 moderate-to-severe VCF events.
11	Explain handling of quantitative	Methods 2.4–2.7, pp. 5–7.	Quantitative variables were handled as follows: L1–L3 HU averaged at patient level;

	variables; describe groupings chosen		HU-vBMD derived using a QCT-referenced regression equation; AI-vBMD averaged across L1–L3; IMAT ratio averaged across L1–L3; continuous predictors standardized as z scores before modeling; VCF status grouped as Genant <2 vs. Genant ≥2.
12(a)	Describe all statistical methods, including those used to control for confounding	Methods 2.7 Statistical analysis, pp. 6–7.	Statistical methods include group comparisons, Pearson correlation, ICC, Bland–Altman analysis, logistic regression, ROC/AUC analysis, paired DeLong tests, Youden-optimal thresholds, sensitivity, specificity, PPV, NPV, Brier score, Hosmer–Lemeshow test, bootstrap internal validation, collinearity diagnostics for M5, and decision curve analysis. Confounding was addressed in M5 by including age, BMI, adjuvant endocrine therapy, and radiotherapy.
12(b)	Describe any methods used to examine subgroups and interactions	N/A	No subgroup or interaction analyses were prespecified or reported.
12(c)	Explain how missing data were addressed	Methods 2.1 and 2.7, pp. 3 and 6–7; Table 1 footnote, p. 8.	Patients with incomplete QCT or missing key clinical data were excluded; complete-case analysis was performed. Table 1 states that among the 275 patients in the final complete-case cohort, no missing data were present for the variables analyzed.
12(d)	If applicable, describe analytical methods accounting for sampling strategy	N/A	This was a single-center consecutive retrospective cohort; no complex sampling strategy was used.
12(e)	Describe any sensitivity analyses	N/A	No sensitivity analyses were reported.
13(a)	Report numbers of individuals at each stage (e.g., eligibility, included)	Methods 2.1, pp. 3–4; Figure 1, p. 4; Results 3.1, pp. 7–8.	Numbers at each stage are reported: 311 screened, 36 excluded, 275 included in the final analytic cohort, 75 with Genant grade ≥2 VCFs, and 200 with Genant grade <2.
13(b)	Give reasons for non-participation at each stage	Methods 2.1 Participants, pp. 3–4; Figure 1, p. 4.	Reasons for exclusion are reported: poor CT image quality, vertebral implants, incomplete QCT results, and missing key clinical information.
13(c)	Consider a flow diagram	Figure 1, p. 4.	Patient selection and analytic cohort flow diagram is provided. The diagram reports screened patients, exclusions with reasons, final analytic cohort, VCF status, index assessments, and model analyses.
14(a)	Give characteristics of participants and	Results 3.1, pp. 7–8; Table 1, p. 8.	Participant characteristics are reported, including age, BMI, adjuvant endocrine therapy, radiotherapy, QCT-vBMD, HU-

	information on exposures		vBMD, AI-vBMD, and paraspinal IMAT ratio stratified by VCF status.
14(b)	Indicate number of participants with missing data for each variable of interest	Methods 2.1 and 2.7, pp. 3 and 6–7; Table 1 footnote, p. 8.	Incomplete QCT or key clinical data were exclusion criteria. Complete-case analysis was used. No missing data were present for variables included in the final complete-case cohort.
15	Report numbers of outcome events or summary measures	Results 3.1, pp. 7–8; Figure 1, p. 4.	Outcome events are reported: moderate-to-severe VCF, defined as Genant grade ≥2, was present in 75/275 patients (27.3%); 200/275 patients (72.7%) had Genant grade <2.
16(a)	Report unadjusted (and if applicable adjusted) estimates with precision (95% CI)	Results 3.2–3.4, pp. 8–13; Tables 2–4, pp. 9 and 12–13; Figures 2–4, pp. 9–12.	Estimates with precision are reported, including Pearson r with 95% CI, ICC with 95% CI, Bland–Altman limits of agreement, ORs with 95% CI, AUCs with 95% CI, DeLong p values, Brier scores, Hosmer–Lemeshow p values, decision curve analysis, and bootstrap internal validation results in Supplementary Table S3. Table 2 and Figure 2 show agreement analyses and Table 4 summarizes model performance.
16(b)	Report category boundaries when continuous variables were categorized	Methods 2.6 and 2.7, pp. 6–7.	Genant grades 2–3 were defined as moderate-to-severe VCFs. Continuous predictors were standardized as z scores and were not categorized for logistic regression modeling.
16(c)	If relevant, translate estimates of relative risk into absolute risk	N/A	The study focuses on diagnostic performance and cross-sectional identification rather than relative risk estimation; translation of relative risk into absolute risk was not applicable.
17	Report other analyses (e.g., subgroups, interactions)	Results 3.3 and 3.4, pp. 10–13; Figures 3–4, pp. 11–12; Tables 3–4, pp. 12–13; Supplementary Tables S1–S4.	Other analyses include inter-reader reliability for Genant grading, paired DeLong model comparisons, Youden-optimal thresholds, bootstrap internal validation, collinearity diagnostics, and decision curve analysis. No subgroup or interaction analyses were reported.
18	Summarize key results with reference to objectives	Discussion, first paragraph, p. 13.	Key results are summarized with reference to study objectives: agreement with QCT-vBMD, independent association of paraspinal IMAT ratio with VCF, incremental value of adding IMAT ratio to AI-vBMD, and highest performance of the imaging-clinical model.
19	Discuss limitations and their direction/magnitude	Discussion, limitations paragraph, pp. 15–16.	Limitations are discussed, including retrospective single-center design, possible selection of a highly characterized subgroup due to the non-contrast CT and QCT-within-

			7-days requirement, prevalent rather than incident VCF endpoint, incomplete treatment and patient-level factors, L1–L3 rather than full thoracolumbar quantification, cohort-specific HU-vBMD calibration, software-version specificity, inaccessible proprietary thresholds and segmentation rules, need for broader validation, cautious interpretation of individual clinical covariates, and apparent M4 calibration misfit.
20	Provide a cautious overall interpretation considering objectives, limitations, multiplicity, similar studies, other evidence	Discussion, pp. 13–16; Conclusions, p. 16.	Interpretation is cautious and considers objectives, related evidence, and limitations. The manuscript emphasizes cross-sectional identification rather than prospective fracture prediction, avoids causal interpretation of treatment associations, and frames the approach as a quantitative adjunct rather than a replacement for radiologist assessment or dedicated densitometry.
21	Discuss the generalisability (external validity) of the study results	Discussion, limitations paragraph, pp. 15–16.	Generalizability is discussed. Broader validation across CT vendors, reconstruction settings, scanner platforms, software implementations, and patient populations is stated as necessary. The manuscript also notes that the requirement for both true non-contrast CT and lumbar QCT within 7 days may limit generalizability to broader breast cancer imaging populations.
22	Give the source of funding and the role of the funders	Funding statement, p. 16.	Funding sources and funder roles are reported: National Key R&D Program of China (grant no. 2020YFA0714002) and Master's Research Innovation Project of the First Clinical College of Chongqing Medical University (no. CYYY-SSCX202525). The funders had no role in study design, data collection, analysis, interpretation, manuscript writing, or decision to publish.
